# Arm swing asymmetry in overground walking

**DOI:** 10.1038/s41598-018-31151-9

**Published:** 2018-08-24

**Authors:** Tim Killeen, Morad Elshehabi, Linard Filli, Markus A. Hobert, Clint Hansen, David Rieger, Kathrin Brockmann, Susanne Nussbaum, Björn Zörner, Marc Bolliger, Armin Curt, Daniela Berg, Walter Maetzler

**Affiliations:** 1 Spinal Cord Injury Center, University Hospital Balgrist, Zurich, Switzerland; 2 Department of Neurology, University Hospital Schleswig-Holstein, Campus Kiel, University of Kiel, Kiel, Germany; 3 Center for Neurology and Hertie Institute for Clinical Brain Research (HIH), Department of Neurodegeneration, University of Tübingen, Tübingen, Germany; 4 DZNE, German Center for Neurodegenerative Diseases, Tübingen, Germany; 5 Department of Neurology, University Hospital Zurich, Zurich, Switzerland

## Abstract

Treadmill experiments suggest that left-dominant arm swing is common in healthy walking adults and is modulated by cognitive dual-tasking. Little is known about arm swing asymmetry in overground walking. We report directional (dASI) and non-directional arm swing symmetry indices (ndASI) from 334 adults (mean age 68.6 ± 5.9 y) walking overground at comfortable (NW) and fast (FW) speeds and while completing a serial subtraction task (DT). dASI and ndASI were calculated from sagittal shoulder range of motion data generated by inertial measurement units affixed to the wrist. Most (91%) participants were right-handed. Group mean arm swing amplitude was significantly larger on the left in all walking conditions. During NW, ndASI was 39.5 ± 21.8, with a dASI of 21.9 ± 39.5. Distribution of dASI was bimodal with an approximately 2:1 ratio of left:right-dominant arm swing. There were no differences in ndASI between conditions but dASI was smaller during DT compared to FW (15.2 vs 24.6; p = 0.009). Handedness was unrelated to ndASI, dASI or the change in ASI metrics under DT. Left-dominant arm swing is the norm in healthy human walking irrespective of walking condition or handedness. As disease markers, ndASI and dASI may have different and complementary roles.

## Introduction

Rhythmic swinging of the arms is a universal feature of human bipedal gait that appears to have a minor^1–4^ or negligible^5^ role in reducing the metabolic cost of walking and is likely subject to a mix of cortical^6,7^ and lower-level^1,8–10^ neural control. Asymmetry of arm swing during gait is often observed in Parkinson’s disease (PD) and may be present in the prodromal stage of the disorder^11–14^. Asymmetrical arm swing nevertheless seems to be a common feature of otherwise normal gait^7,15,16^. Interestingly, studies in healthy individuals consistently report left arm swing amplitudes greater than those on the right^7,15–17^.

Why arm movements should be greater on the left is unclear, but a logical candidate mechanism would be a discrepant degree of automatism of the neural apparatus controlling arm swing habituated through arm preference for voluntary tasks, i.e. handedness. If this is the case, then the ubiquity of left-dominant arm swing may simply reflect the prevalence of right-handedness in our species. Kuhtz-Buschbeck *et al*. observed treadmill walking in eight right-handed and eight left-handed individuals and reported left-dominant arm swing in both groups^16^, concluding that asymmetry is not related to handedness. Other studies of arm swing during treadmill walking included too few left-handers to confirm these findings^7,15^. To our knowledge, no studies have evaluated directional (i.e. the degree of left- or right-dominant) arm swing asymmetry indices (ASI, see Methods and Supplementary Fig. 1) in overground walking. Clarification of the relationship between ASI and arm dominance would facilitate the development of arm swing metrics as markers of movement disorders and may inform models of the neural control of arm swing and gait in general.

Increasingly, dual-task paradigms are being used with the aim of increasing the sensitivity of gait analysis in the detection of parameters indicative of impaired cognitive-motor functioning, which is associated with aging, falling and disease states^18–22^. Arm swing asymmetry has been identified as a potential prodromal biomarker for falls and PD^11–13^. Methodologies are heterogenous and most authors report non-directional ASI (ndASI), agnostic to the directional component of the asymmetry.

In the few studies reporting directional ASI (dASI), certain cognitive dual-tasks appear to exert a lateralised influence on arm swing, with the left-lateralised Stroop language task reducing right arm swing and enhancing left-dominant asymmetry^7,15,17^. Little is known, however, about the effect of the well-established serial subtraction task^23^ on dASI. Mirelman *et al*. observed a dramatic increase in ndASI during serial subtraction in older adults^24^, although Plate *et al*. noted no effect of counting backwards on dASI and ndASI in their cohort of healthy adults while reporting a significant increase in both forms of ASI during a Stroop dual-task. A characterisation of the effect of serial subtraction, widely-utilised in clinical practice and research, on both dASI and ndASI in target cohorts walking overground is lacking.

This study provides norm data for arm swing symmetry in a large cohort of older adults ambulating overground under normal walking, fast walking and serial subtraction dual-task conditions. We assess the influence of increased walking speed and cognitive distraction and analyse the demographic factors, including handedness, as well as PD prodromal factors, that may be associated with this novel metric. Insights from this study may provide the basis for future investigation of arm swing metrics as diagnostic and treatment response markers in movement disorders.

## Methods

This study used cross-sectional data from the follow-up cohort of the TREND study (Tübinger evaluation of Risk factors for Early detection of NeuroDegeneration)^25^. Participants aged 50–85 years were recruited via newspaper announcements and public events. Exclusion criteria included a diagnosis of a neurodegenerative disorder, stroke, inflammatory CNS disease (including multiple sclerosis, meningitis and vasculitis) or medication with antipsychotics or antidopaminergic drugs. Participants had to be able to walk without aids and have no significant hearing or visual impairment. A neurological examination was performed and individuals with evidence of peripheral polyneuropathy were excluded from this analysis. The study was carried out in accordance with the Declaration of Helsinki and Good Clinical Practice guidelines and was approved by the ethical committee of the medical faculty of the University of Tübingen (90/2009BO2). All subjects gave written, informed consent.

Participants were instrumented for gait analysis with six inertial measurement units (IMU; Mobility Lab, APDM, USA), comprising trunk, lumbar and bilateral ankle sensors and one fixed to each wrist dorsally with a Velcro® strap. The 4.8 × 1.3 × 3.6 cm IMUs weigh 22 g and output activity counts at a sampling frequency of 128 Hz. Range of motion (RoM) data for each arm was calculated in degrees by the software (Mobility Lab, v.1.0.0.201410210356, APDM, USA) using a proprietary algorithm. As we were only interested in steady-state ambulation, data for the turns as well as the acceleration and deceleration phases at each end of the walkway were filtered out and excluded from the analysis as described by Hollmann and colleagues^26,27^.

Participants performed three walking tasks in a well-lit, 2 m-wide hallway without obstacles or handrails. For all tasks, participants were required to walk 20 m back and forth with a turn at each end for one minute. Participants could choose with which foot they began the assessment and no specific instructions were given as to how or in which direction participants should turn. In the first task (normal walking), participants were asked to walk at a self-selected, “comfortable pace”. During the fast walking task, they were asked to walk “as fast as possible, but not running and not endangering yourself”. In the third task, participants walked at the same, fast pace but were additionally required to perform a serial subtraction task, counting down in 7 s from 408 continuously for the whole minute. For the latter task, participants were explicitly asked to perform both aspects of the dual task (walking and subtracting) without prioritising one at the expense of the other.

Arm swing RoM data were calculated and exported using the Mobility Lab software and these outputs reorganised into Excel 2010 (Microsoft, Redmond, WA, USA) using a short MATLAB (R2016a; The Mathworks, Natick, MA, USA) script. Arm swing asymmetry can be described as a non-directional index (ndASI) or using a directional, absolute arm swing symmetry index (dASI)^7,14,15,28^, where positive values indicate proportionally greater movements on the left and *vice versa*. Both arm swing indices were calculated from the left and right arm RoM values as follows (see also Supplementary Fig. 1):

$$ndASI=ABS\,(\frac{L-R}{max(L,R)})\times 100$$

in which L is the sagittal range of motion on the left and R that on the right. In contrast, dASI allows conclusions about lateralised effects to be drawn:

$$dASI=\,(\frac{L-R}{max(L,R)})\times 100$$

For the dASI, the convention that left dominant arm swing yields positive values was adhered to.

Statistical analysis was performed using SPSS 24.0 (IBM Corp, Armonk, NY, USA) and Prism v. 7.0.3 (Graphpad Software, La Jolla, CA, USA). Population frequencies of dASI were plotted and non-linear regression models tested for goodness of fit. Arm swing parameters under each condition were analysed with a linear mixed model (LMM) in which condition (NW, FW, DT) was a repeated measure. Demographic fixed effects comprised gender, age, weight, height and handedness. Handedness was categorised into five groups based on the Edinburgh Handedness Inventory in line with previous work^29^. Participants with an EHI score between 70 and 100 were deemed strongly right-handed, 20–69 mixed right handers, −20–20 of mixed handedness, −70–−20 mixed left-handers and −100–−70 strongly left-handed. In addition, habitual walking speed, fall status (self-reported fall within the last 6 months), PD prodromal factor status (one of hyposmia, depression, rapid eye movement behaviour disorder^30^), years of formal education, Montreal Cognitive Assessment (MoCA) score, time taken for completion of the preparatory serial subtraction task while standing and the cognitive dual-task cost (DTC). The DTC was calculated by subtracting the corrected response rate (CRR; the response rate per second multiplied by the percentage of correct responses) during the walking dual-task from the CRR during the single-task serial subtractions while standing and then dividing the result by the CRR without the dual task^31^. To assess condition effect on arm RoM itself, a mixed, two-way ANOVA was performed with condition (repeated measure) and arm (left or right) as factors. Post-hoc t-tests were performed where indicated, with Bonferroni correction for multiple comparisons.

## Results

A total of 334 individuals (176 males; 52.7%) were included in this analysis. Mean age at enrolment was 68.6 ± 5.9. Further demographic details are available in Table 1. Forty-six individuals (14.6%) reported at least one fall in the last 6 months and 116 (34.7%) had at least one prodromal marker for PD. Three hundred and four (91%) participants were either strong or mixed right-handers, while 26 (7.8%) were strong or mixed left-handers.

**Table 1:** Characteristics of the cohort. SD: standard deviation, EHI: Edinburgh Handedness Inventory, MoCA: Montreal Cognitive Assessment, DT: dual-task; prodromal factors: hyposmia, depression, rapid eye movement behaviour disorder.

	n	Mean	SD
Age (years)	334	68.6	5.9
Height (cm)	334	1.69	0.09
Weight (kg)	334	78.2	15.4
Handedness (assessed with EHI laterality index; %)	334	77.1	38.9
Strong right-handers	255 (76.3%)	94.2	8.8
Mixed right-handers	49 (14.7%)	54.8	12.5
Mixed handedness	4 (1.2%)	15.9	4.7
Mixed left-handers	23 (6.9%)	−34.1	18.5
Strong left-handers	3 (0.9%)	−76.3	5.5
MoCA score	334	25.1	3.0
Years of Education	334	14.2	2.8
Normal walking speed (m/s)	334	1.29	0.17
Fast walking speed (m/s)	334	1.57	0.25
DT walking speed (m/s)	332	1.30	0.22
Time taken for ten serial subtractions (preparation test, standing; sec)	332	38.0	17.2
Number of mistakes in subtraction task (preparation test, standing)	332	0.74	1.12
Number of mistakes in subtraction task (while walking)	332	1.53	1.64
Cognitive dual-task cost	332	0.42	0.24
Categorical variables	n	percentage	
Prodromal factors present	116	34.7	
1 factor present	103	30.8	
2 factors present	9	2.7	
3 factors present	4	1.2	
Fall reported in last 6 months	46	14.6	
Male gender	176	52.7

**Table 2:** Results of the linear mixed model with walking condition as a repeated measure. ASI: arm swing symmetry index, PD: Parkinson disease, MoCA: Montreal Cognitive Assessment score.

Factor	Directional ASI		Non-directional ASI	
	F	p	F	p
Intercept	1.106	0.293	8.847	**0**.**003**
Age	3.660	0.056	0.047	0.828
Height	0.125	0.724	2.130	0.145
Weight	0.866	0.352	0.362	0.548
Handedness	1.145	0.334	0.636	0.637
MoCA	0.660	0.417	0.679	0.410
Years of education	3.825	0.051	0.016	0.900
Walking Speed	0.998	0.381	1.264	0.261
Time required for cognitive task while standing	0.597	0.440	0.818	0.366
Cognitive dual-task cost	0.772	0.380	0.014	0.907
PD prodromal factor	0.240	0.624	4.845	**0**.**028**
Faller status	2.666	0.103	1.202	0.273
Walking condition	4.726	**0**.**009**	0.198	0.820
Gender	0.911	0.340	7.640	**0**.**006**

Mean habitual walking speed was 1.29 m/s (4.64 km/h). Mean walking speed during fast walking without dual task was 1.57 m/s (5.65 km/h), whereas fast walking with cognitive task lead to a significant reduction of walking speed to 1.30 m/s (4.68 km/h). The cognitive DTC while walking and performing the serial subtraction task was 0.42 ± 0.24, indicating worse performance during the dual-task compared to completing the same task while standing.

**Figure 1:**
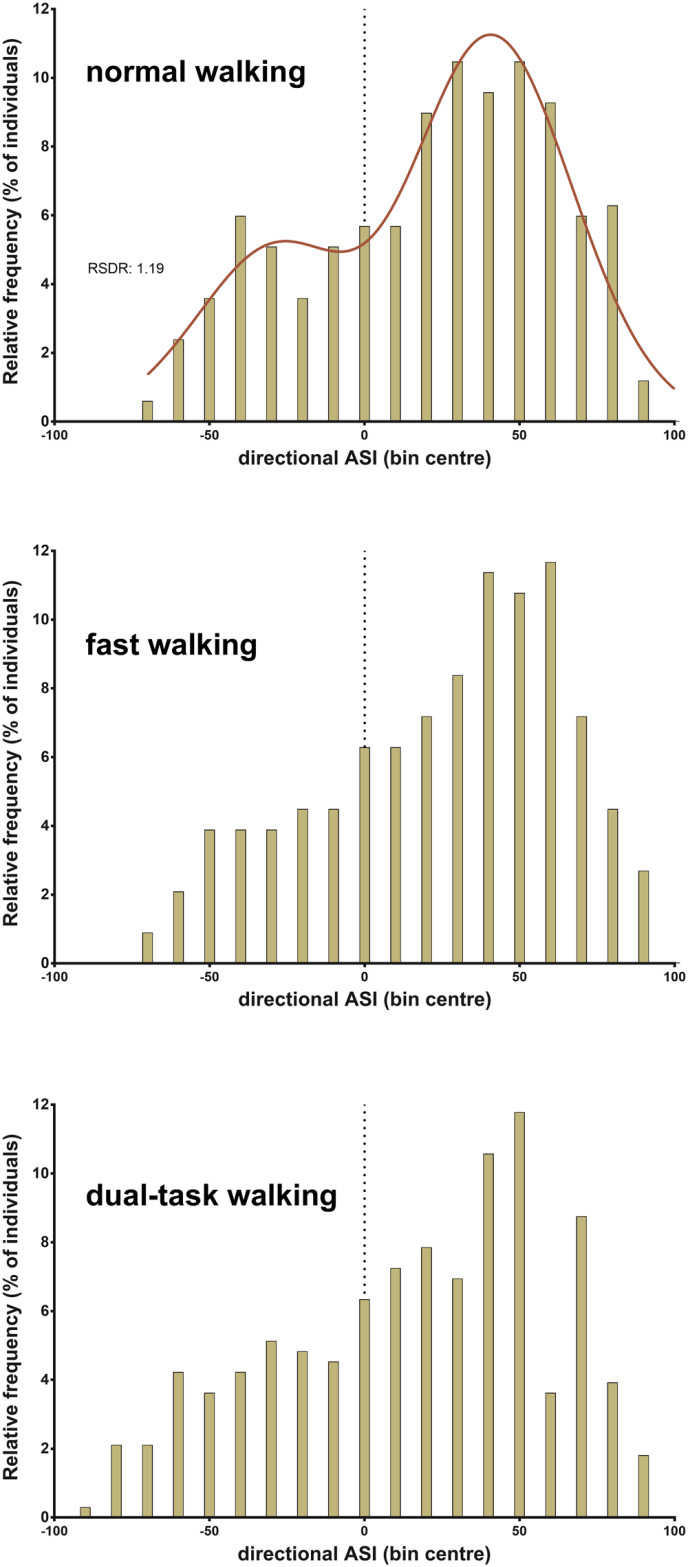
Directional arm swing symmetry index (dASI) frequency distributions for the individual walking conditions as indicated. The red line shows a bimodal Gaussian regression model which best explained the frequency data for normal walking with mean values at −25 and +42 dASI. The dotted line indicates dASI of 0, at which arm swing amplitudes are symmetrical. Positive values indicate left-dominant arm swing asymmetry and *vice versa*. RSDR: robust standard deviation of the residuals. For normal walking and fast walking, n = 334, for dual-task walking, n = 332.

**Figure 2:**
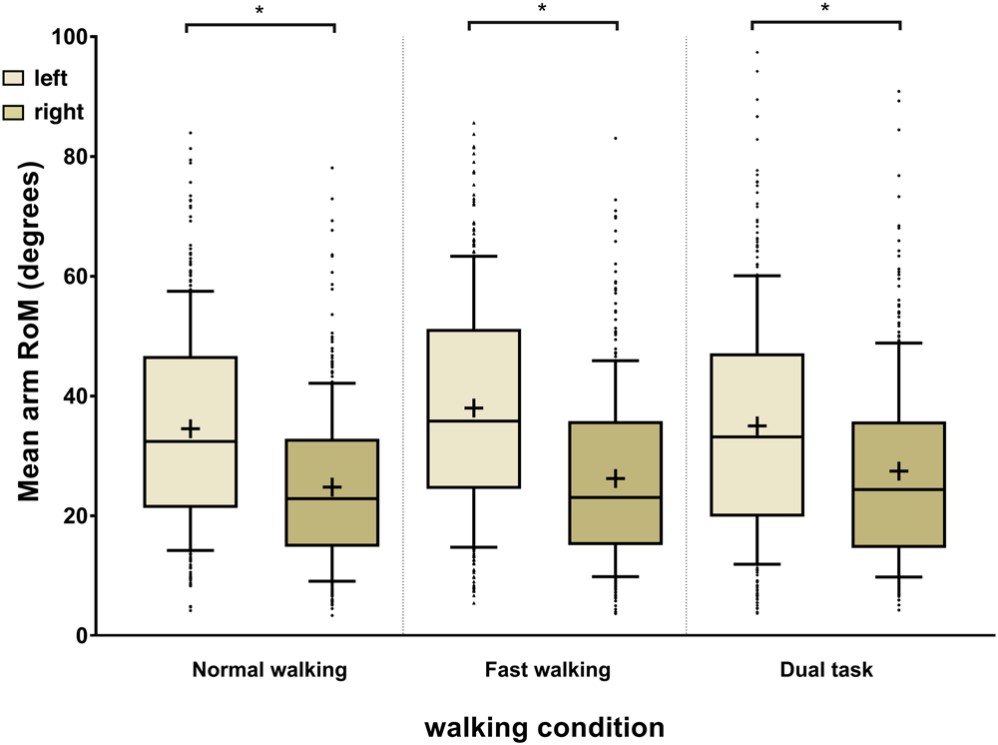
Left and right arm swing amplitudes under the three walking conditions (Tukey box-plots), ^+^indicates the mean. *Indicates significance at the p ≤ 0.05 level (mixed ANOVA, condition as repeated measure). RoM: range of motion. For normal walking and fast walking, n = 334, for dual-task walking, n = 332.

Arm swing RoM data was available for all participants in the normal walking and fast walking tasks. In the dual-task condition, data was not available for two individuals who were unable to perform the task. Asymmetrical arm swing was highly prevalent in the cohort during normal walking, with a mean ndASI of 39.5 ± 21.8 and a dASI of 21.9 ± 39.5 indicating a marked preponderance of left-dominant arm swing. The frequency of dASI values during normal walking was best explained by a bimodal sum of two Gaussian distributions with means at dASI = −25 and dASI = 42 (Fig. 1). Two hundred and fifty-seven participants (77.0%) displayed baseline dASI values of greater than 20 (n = 193; 57.8%) or less than −20 (n = 64; 19.2%). Those with dASI values greater than 50 (n = 95; 28.4%) or less than −50 (n = 14; 4.2%) comprised 32.6% of the cohort. Mean arm swing amplitude was significantly larger on the left than on the right in all walking conditions (Fig. 2).

Overall, ASI was reproducible within subjects, with 250 of 332 individuals (75.3%) maintaining the same direction of arm swing asymmetry across all three locomotor modes.

**Figure 3:**
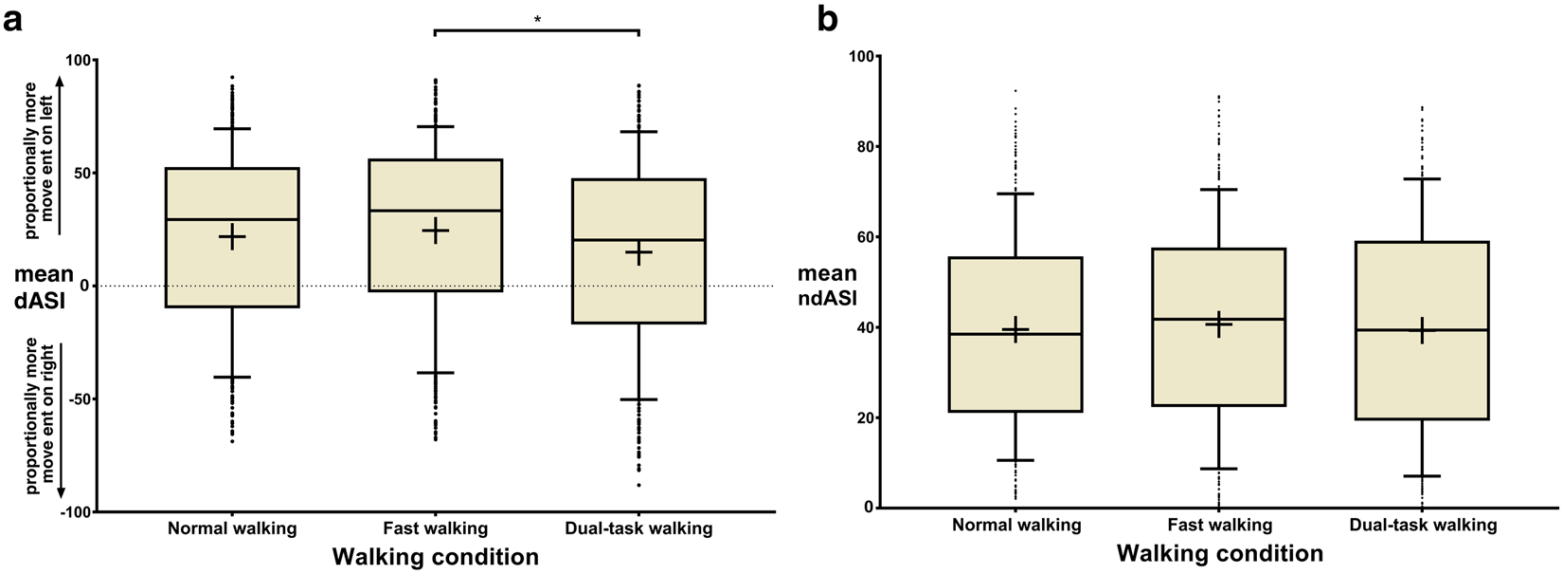
(**a**) Effect of walking condition on directional arm swing asymmetry index (dASI), where *indicates significance at the p ≤ 0.05 level (post-hoc paired t-tests with Bonferroni correction for multiple comparisons). The dotted line indicates a dASI of 0, where arm swing amplitudes are symmetrical. (**b**) Effect of walking condition on non-directional arm swing asymmetry index (ndASI). For normal walking and fast walking, n = 334, for dual-task walking, n = 332.

The only covariate significantly affecting dASI was walking condition (p = 0.009), although years of education (p = 0.051) and age (p = 0.056) approached significance (Table 2). Pairwise comparison of the walking conditions revealed that mean dASI was only affected by dual-task walking as compared to fast walking (15.2 vs 24.6; p = 0.009; Fig. 3a).

Individuals with left-dominant arm swing (dASI > 20) during normal walking responded particularly strongly to the dual-task, with a significant decrease in their baseline, left-dominant asymmetry compared to the other tasks (p ≤ 0.018). Those with right-dominant arm swing (<−20) showed no significant changes in dASI under the different tasks. The same pattern was observed for those with highly asymmetrical baseline arm swing; participants with a baseline dASI of >50 significantly decreased their dASI under the dual-task to walk with more symmetrical arm swing (p ≤ 0.004). The change in dASI between the walking conditions for all participants is presented in Supplementary Fig. 2.

**Figure 4:**
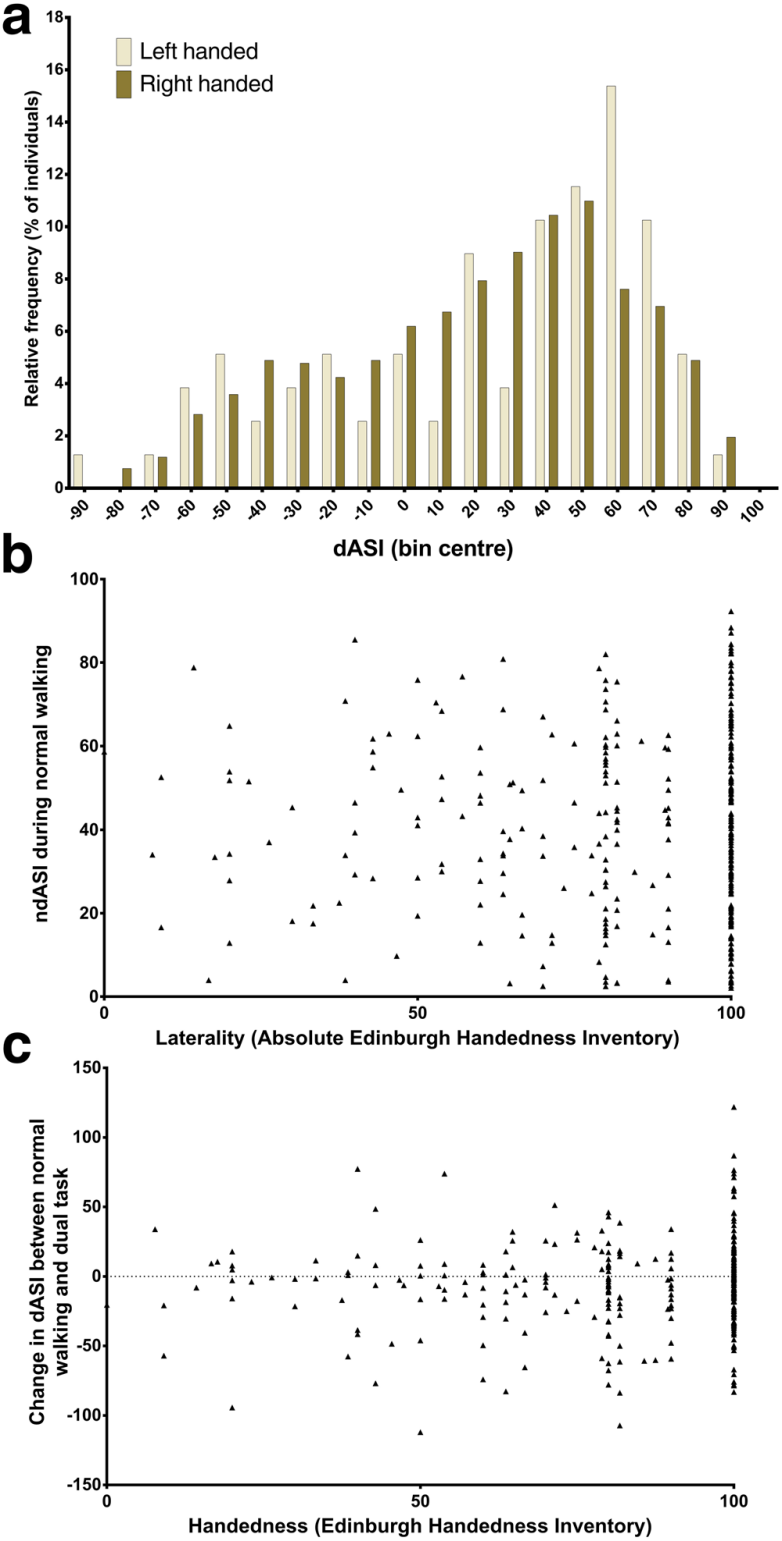
Relationship between handedness and arm swing symmetry index. (**a**) Relative frequency distribution of left- and right-handers. (**b**) scatterplot of dASI vs Edinburgh Handedness Inventory Laterality Index (EHI), showing no association between the metrics. Likewise, when EHI and the change in dASI under dual-task conditions are plotted (**c**), no relationship is seen.

There was no difference in either ASI metric between any of the handedness groups in any of the three walking tasks, nor for left- and right-handers dichotomised at EHI = 0 (Fig. 4). Handedness was also not associated with changes in dASI between normal walking and either of the other tasks. Similarly, there were no correlations between handedness expressed as an absolute laterality index (akin to the ndASI) and ndASI (Supplementary Fig. 3) or changes in dASI or ndASI between conditions.

For ndASI, no condition effect was observed (p = 0.820). However, gender (p = 0.006) and the presence of one or more PD prodromal factor (p = 0.028) had significant effects in the model (Table 2). Post-hoc analyses revealed that females exhibited significantly less overall arm swing asymmetry than males during the dual-task (ndASI 41.4 vs 37.7; p = 0.001).

## Discussion

This is the first study to systematically report directional arm swing asymmetry in a large cohort of healthy older walkers. A large degree of asymmetry is evidently the norm in human overground locomotion with the mean dASI of 21.9 somewhat larger than values reported in the treadmill literature of 6.6–18.0^7,15–17^. As a metric, dASI proved to be consistent within subjects, with three quarters of participants maintaining the direction of asymmetry across the three tasks, somewhat higher than the 63% reported by Kuhtz-Buschbeck *et al*. in their treadmill-based study^16^. These data, generated from relatively cheap and easily-applied IMUs, confirm that 3D kinematic gait analysis may not always be necessary for the study of ASI in movement disorders. Whether an IMU-based approach can be applied in the clinical setting, where a high degree of individual measurement precision is often required, is less clear and requires further investigation.

Multiple authors have now reported left-dominant arm swing asymmetry during normal walking, as well as under a variety of other walking conditions on the treadmill and overground^7,15,24,32^. Using a large cohort, including 26 left-handers, this study goes further than previous work^7,16^ in conclusively refuting an association between handedness and ASI. These comprehensive data are not compatible with the hypothesis that growing up left- or right-handed and thus selectively habituating one upper limb to corticospinally-controlled, fine-motor tasks, results in reduced ipsilateral arm swing amplitude – be it through reduced ipsilateral automaticity during gait or other putative mechanisms.

Beyond handedness, many human behaviours with various degrees of automatism exhibit lateral biases. Some, such as an aesthetic preference for viewing scenes with light sources to the upper left^33^, are associated with handedness. In such cases, left-handers do not display a mirrored response to right-handers by preferring right-lit scenes, instead exhibiting the same left-lit bias to a significantly lesser degree. Left-handers grow up in a right-hander’s world and are constantly exposed to environments and objects designed to be approached or manipulated with the right hand and, consequently, innate tendencies to mirror right-handers’ biases may be blunted with experience. Handshakes and microwave doors are examples of environmental dextral conventions which may conceivably promote left-dominant arm swing asymmetry in left- and right handers alike, with the right arm “primed” for action^34,35^. If such a cultural override of innate right-dominant arm swing in left-handers is the case, it must be remarkably strong, as left- and right-handed populations exhibited identical phenotypes in our study. Presumably, such an effect would develop with experience of living in a right-handed environment, so studies of arm swing in the gait of left-handed children and adolescents, currently entirely lacking, would be informative.

This “cultural” account does not, however, easily explain the observed bimodal distribution of ASI in our sample, in which a significant minority of both left- and right-handers consistently demonstrated right-dominant arm swing (a ratio of approximately 2:1 left:right dominant swing). Arm swing asymmetry may, instead, be more closely related to the evolutionarily older, innate laterality biases typified by rotational behaviour^36^. When prompted to turn, children and adolescents do so counterclockwise 59–79% of the time^37^, with no clear correspondence to handedness^38,39^, while adults demonstrate a 2:1 ratio in favour of both leftward body turns^40^ and turning the head to the right while kissing^41,42^. This 2:1 ratio has been described as characteristic of behavioural asymmetries in animals and humans distinct from the special case of human handedness, which exhibits a 9:1 ratio^43^; for a review see Schaafsma *et al*.^36^. The genesis of these tendencies are not fully understood, but prenatal positioning is thought to be important in animals^36,44^. The finding that female gender was associated with significantly less ndASI in our sample is consistent with evidence suggesting a tendency to more strongly lateralised behaviour in males^7^.

Although we used strict and established methods to exclude acceleration, deceleration and turning between walking bouts in our sample^26^, the possibility remains that turning during overground gait analysis may have influenced dASI. Similar dASI from various treadmill studies^7,15,16^ argue against this interpretation, as do findings from an actimeter study which revealed a 2:1 ratio of left-dominant arm movements over 48 hours of everyday activity^35^, possibly due to arm swing asymmetry and suggesting that the consistent findings from laboratory-based gait analyses may be generalisable to everyday ambulation.

Arm swing and its asymmetry are attractive as gait analysis metrics as they are comparatively easy to measure and abnormalities of arm swing may manifest early and unilaterally in disease states^11–14,17^. It is ubiquitous to, yet non-critical for^2^, normal gait and, as such, may be less subject to voluntary or involuntary prioritisation by patients with mild gait disorders. Unfortunately, high inter-individual variability makes the development of clinically useful metrics and meaningful cut-off values difficult, irrespective of whether ASI is based on 3D wrist trajectories or sagittal ROMs^7,11,15,17^. In certain settings, ASI is modulated by cognitive dual-task paradigms, with consistent changes in ASI under cognitive loading seen during walking in healthy aging^7,24^, Parkinson disease^11,15,22^ and incomplete spinal cord injury^17^, potentially rendering ASI more sensitive and thus more useful in diagnosis and measuring responses to treatment in gait disturbance.

Previous work has shown that cognitive dual-tasks must be demanding if they are to evince measurable, consistent changes in gait parameters^15,18,22,45–47^. In this study, the application of an additional cognitive load, in the form of the commonly-used serial sevens subtraction task, resulted in a small yet significant decrease in the prevailing left-dominant asymmetry as measured by dASI compared to fast walking. No condition effect was seen with ndASI. This contrasts with studies using the Stroop task, which invariably enhances the degree of asymmetry and may, through activation of left hemisphere structures, specifically promote left-dominant arm swing^7,15,17^. Previous work looking at the serial sevens or similar mental calculation tasks has generally shown small increases in ndASI measures^11,24^, with only one study directly comparing both dASI and ndASI during simple backward counting^15^. In this unique case, the more cognitively demanding Stroop task elicited more directional and non-directional asymmetry than mental subtraction^15^. Most of these studies were treadmill-based.

The relative simplicity of our serial sevens task in a well-educated cohort (participants had one more year of formal education than the German national average; 14.2 vs 13.2 years^48^) may explain why no increase in non-directional ASI was observed in this study, although mean dual task costs of 42% were in line with those reported previously in older cohorts. Although the mean MoCA score in our cohort, at 25.1, was below that formally defined as indicating mild cognitive impairment (26/30)^49^, large, real-world studies in similar, healthy cohorts reported mean scores of 23–26^50–52^ and suggest that this cut-off value is too strict. Success in serial subtraction tasks is known to be more reflective of calculation skill than concentration^53^ and the participants may not have been sufficiently challenged by the task for much interference with ASI to manifest^24^. The significant shift towards more rightward dASI under the serial sevens task is difficult to account for. Serial subtraction task may act in some individuals as a rhythmic cue, promoting more symmetrical arm swing (i.e. less positive, mean dASI) through entrainment of gait parameters with the articulation of the response. Rhythmic stimuli are able to entrain temporal gait parameters and produce more symmetrical walking in healthy individuals^54^ and PD^55^ and stroke patients^56^. This cuing phenomenon may be stronger when walkers are able to set their own pace in overground, as opposed to treadmill, locomotion, perhaps explaining the divergence of the results in this cohort from those in previous treadmill studies.

Switching from normal walking to the fast walking condition did not influence ndASI or dASI in our cohort, in keeping with prior findings. While arm swing amplitude increases with gait velocity^15,16,24^, left and right tend to do so in concert, resulting in stable ASIs^16^.

In summary, more research is needed into the effect of the commonly-employed cognitive dual-tasks on healthy overground walkers. Standardisation of cognitively-demanding dual-tasks would greatly aid this endeavour. The Stroop task appears to have a stronger and more predictable effect on ASI^15^, particularly when stimulus presentation frequency is pseudorandomised to avoid entrainment effects^7,17^.

Prodromal markers of degenerative movement disorders capable of reliably distinguishing disease from normal aging would be of great utility, allowing early diagnosis and treatment in common, burdensome conditions. Non-directional ASI has formed the basis of initial attempts to develop arm swing metrics of use in diagnosis and treatment response in PD. Here, it was associated with the number of PD prodromal factors in an at-risk cohort representative of clinical practice, although it may be susceptible to gender effects. dASI appears to be more sensitive to aspects related to cognitive-motor interference such as dual-tasking, aging and education. Focussed protocols examining the incidence, evolution and response to dual-tasking of dASI in early PD may result in a useful, cheap and robust metric for early diagnosis. We encourage researchers in this field to report both dASI and ndASI and, for those examining overground walking, to formally record turning tendencies^57^ to better understand the relationship of these lateralised behaviours.

## Conclusion

We report three key findings in this large cohort of relevance to clinicians and researchers investigating movement disorders in the older population. Firstly, a marked degree of left-dominant arm swing is the norm in overground human walking, irrespective of locomotor task. Secondly, these data conclusively reveal handedness to be unrelated to arm swing in healthy populations, suggesting environmental/cultural factors or an unexplained innate laterality bias independent of handedness as possible explanations. Finally, directional ASI is apparently more sensitive to cognitive dual-task effects than its absolute, non-directional counterpart, although different dual-tasks appear to cause different directional responses and the mechanisms and influence of cognitive factors in this response remain unclear. Future research should use cognitively demanding, standardised dual-task paradigms to investigate this interesting parameter which may have utility in diagnosis, monitoring and treatment evaluation in neurodegenerative movement disorders.

## Electronic supplementary material

Supplementary Information
