# Supplementary material for: Arm swing asymmetry in overground walking
Source: Sci Rep. 2018 Aug 24;8:12803. doi: 10.1038/s41598-018-31151-9 (PMC6109135; doi:10.1038/s41598-018-31151-9)
Supplement: Supplementary file 1 — Supplementary Information [file 41598_2018_31151_MOESM1_ESM.docx]

**Arm swing asymmetry in overground walking**

Tim Killeen, Morad Elshehabi, Linard Filli, Markus A. Hobert, Clint Hansen, David Rieger, Kathrin Brockmann, Susanne Nussbaum, Björn Zörner, Marc Bolliger, Armin Curt, Daniela Berg, Walter Maetzler

**Supplementary Materials**

**Supplementary Figure 1**


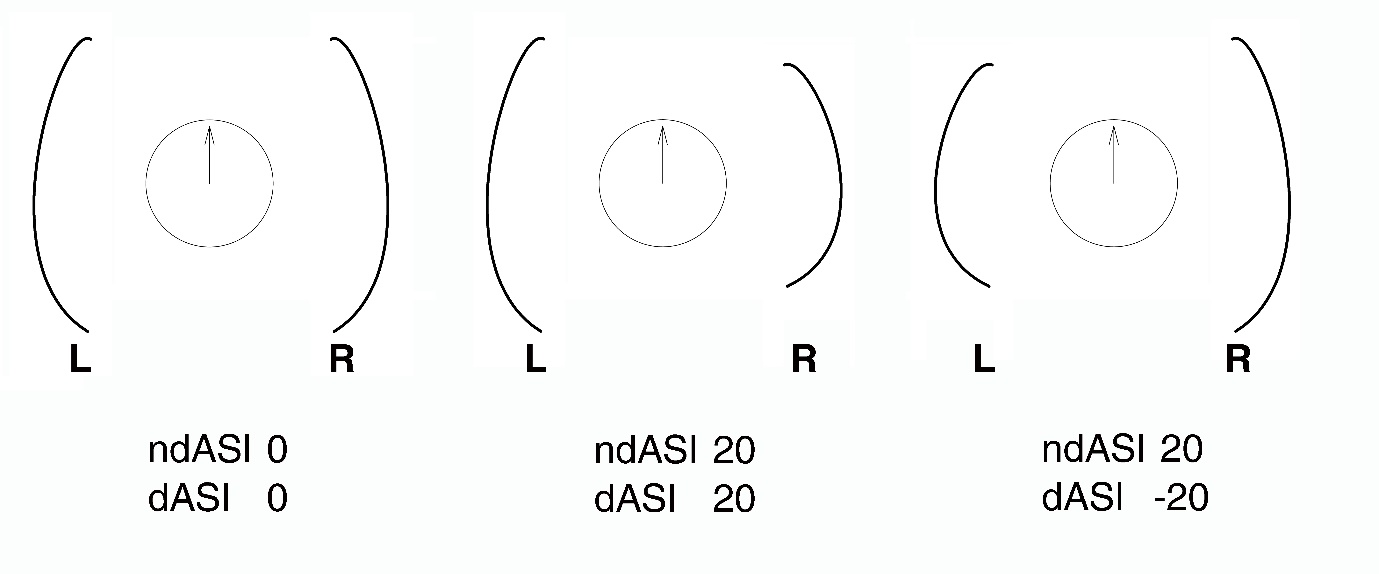
Supplementary Figure 1: Stylised representation of the two arm swing symmetry indices (ASI) used in this paper and how they differ. The images represent a walking individual seen from above in the axial plane; the circle indicates the head and the arrow the direction of progression. The curvilinear black lines represent arm swing trajectories on the left and right. When right arm swing reduces relative to that on the left (b), both indices increase. When the opposite occurs, and left arm swing becomes proportionally smaller than that on the right, ndASI continues to return a positive value and the two states are indistinguishable. The dASI reports a negative value, indicating right-dominant arm swing asymmetry.

**Supplementary Figure 2**


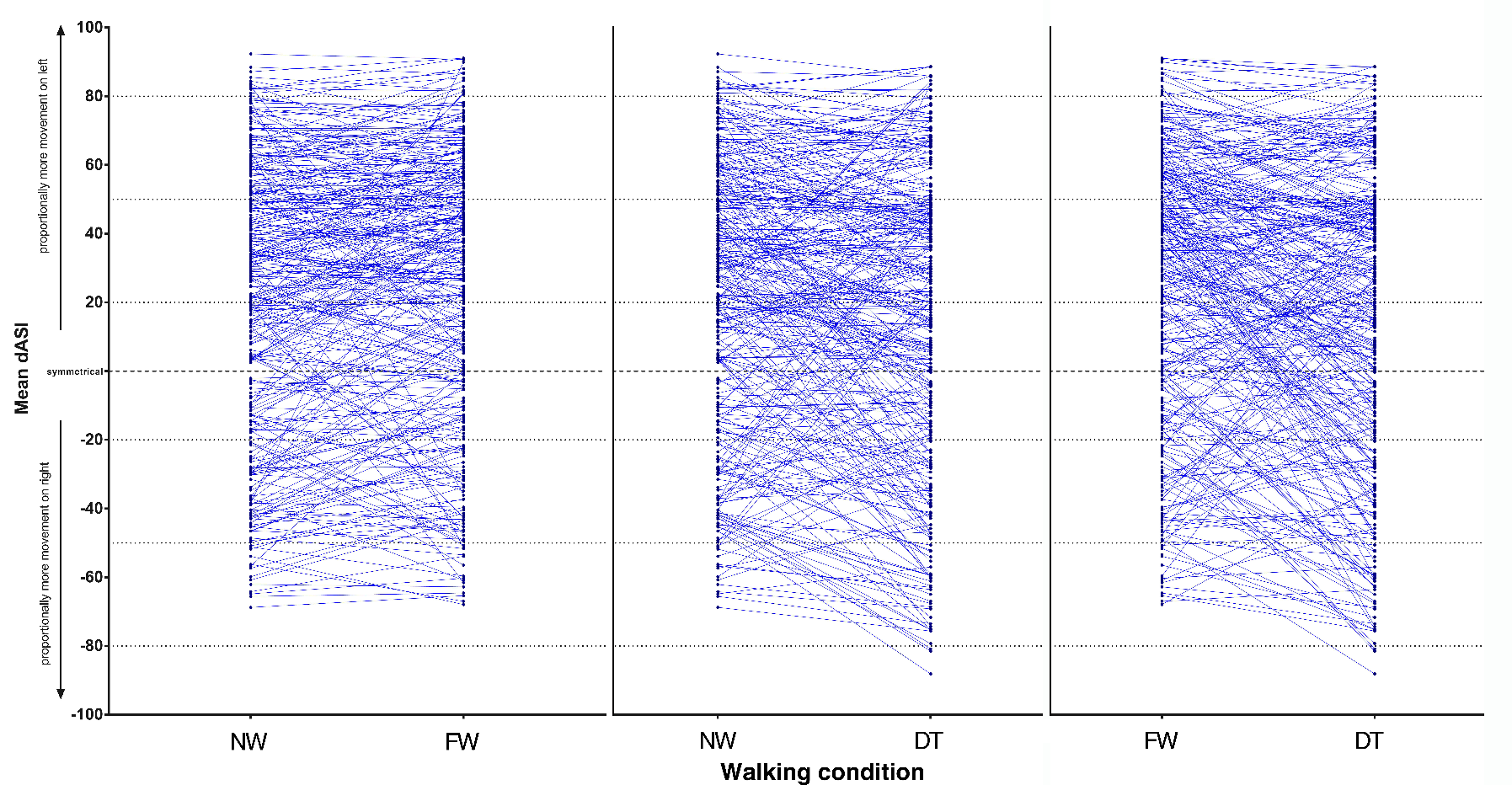


Supplementary Figure 2: Representation of the changes in directional ASI (dASI) for each individual (n=334 for normal walking and fast walking, n=332 for dual-task) under three different walking conditions. NW: normal walking, FW: fast walking, DT: dual-task walking.

**Supplementary Figure 3**

**
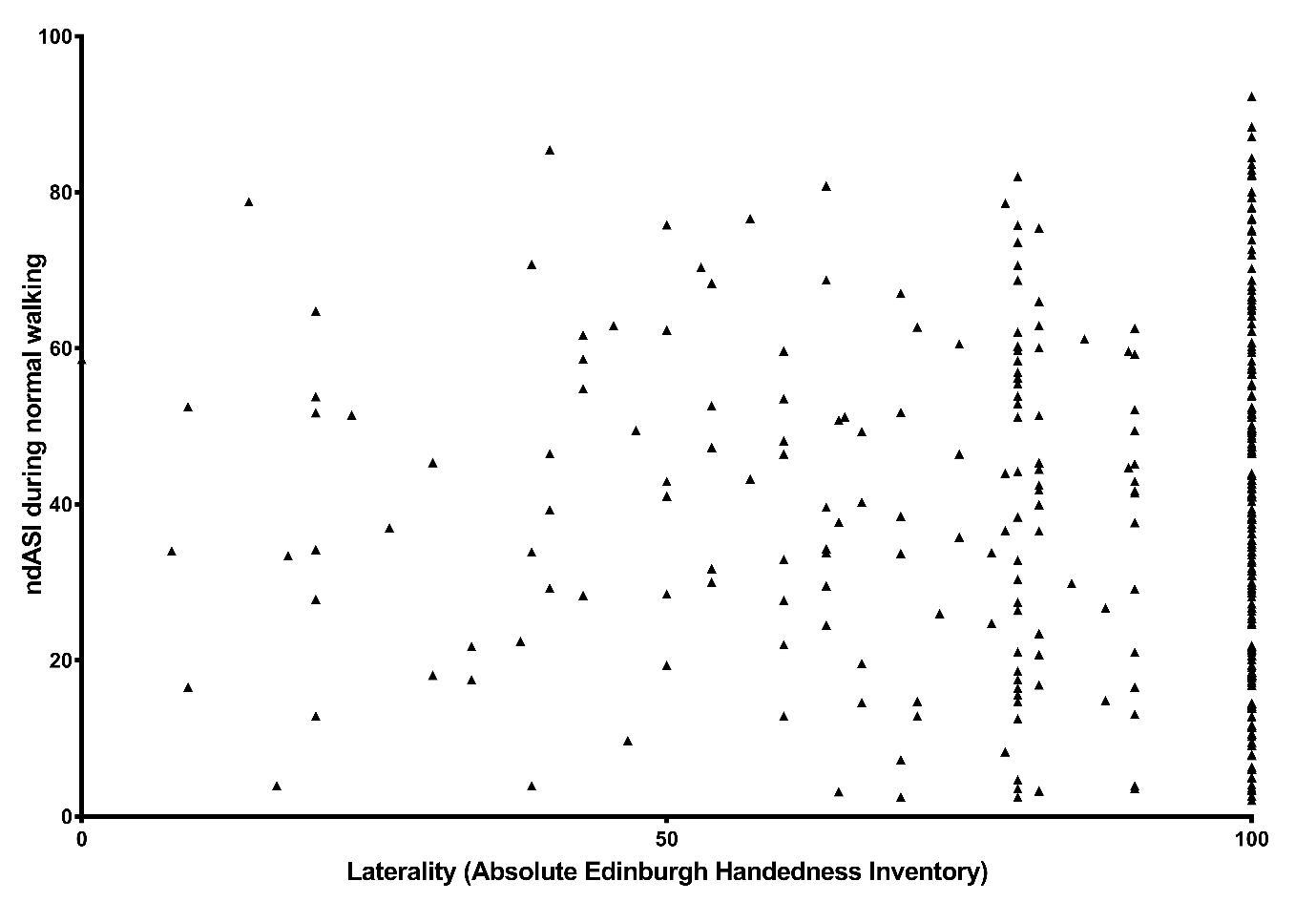
**

Supplementary Figure 3: Relationship between degree of laterality (absolute Edinburgh Handedness Index) and non-directional arm swing symmetry index. Scatterplot shows ndASI vs absolute Edinburgh Handedness Inventory Laterality Index (EHI), with no association between the metrics (Pearson correlation coefficient p=n.s.).
